# Incidence and Site‐Specific Risk Factors of Jaw Osteoradionecrosis for Head and Neck Patients in the IMRT/VMAT Era: Evidence From a Large, Hospital‐Based Cohort

**DOI:** 10.1002/hed.70314

**Published:** 2026-05-10

**Authors:** Fang‐Yu Wu, Hui‐Hsin Ko, Hsiang‐Fong Kao, Shih‐Jung Cheng

**Affiliations:** ^1^ Department of Dentistry National Taiwan University Hospital, College of Medicine Taipei Taiwan; ^2^ Department of Dentistry National Taiwan University Hospital, Jinshan Branch New Taipei Taiwan; ^3^ Graduate Institute of Clinical Dentistry, School of Dentistry, National Taiwan University Taipei Taiwan; ^4^ School of Dentistry, National Taiwan University Taipei Taiwan; ^5^ National Taiwan University Hospital Hsin‐Chu Branch Hsin‐Chu Taiwan; ^6^ Institute of Oncology, National Taiwan University Taipei Taiwan; ^7^ Department of Medical Oncology National Taiwan University Cancer Center Taipei Taiwan; ^8^ Department of Oncology National Taiwan University Hospital Taipei Taiwan; ^9^ Graduate Institute of Oral Biology, School of Dentistry, National Taiwan University Taipei Taiwan

**Keywords:** head and neck cancer (HNC), intensity‐modulated radiotherapy (IMRT), oral cancer, osteoradionecrosis (ORN), radiation dose, risk factors, volumetric‐modulated arc therapy (VMAT)

## Abstract

**Purpose:**

Osteoradionecrosis (ORN) of the jaw remains a serious late complication of head and neck cancer (HNC) radiotherapy. Although intensity‐modulated radiotherapy (IMRT) and volumetric‐modulated arc radiotherapy (VMAT) are expected to reduce ORN risk, large real‐world data in the modern era are limited. This study evaluated the incidence, latency, and risk factors of ORN across radiotherapy modalities in a large hospital‐based cohort.

**Materials and Methods:**

We retrospectively analyzed 6199 patients with HNC treated with curative radiotherapy between 2006 and 2020. ORN was defined as exposed necrotic jaw bone persisting for > 3 months without tumor recurrence. ORN incidence was compared between conventional 2D/3D radiotherapy and IMRT/VMAT. Latency to ORN onset was assessed using Kaplan–Meier analysis, and independent predictors were identified using multivariate Cox regression for demographic, tumor‐related, treatment‐related, and patient‐related variables.

**Results:**

Overall, 278 patients (4.5%) developed ORN. The incidence was 5.5% after 2D/3D radiotherapy and 4.4% after IMRT/VMAT, without a significant overall difference. However, IMRT/VMAT significantly reduced ORN risk in nasopharyngeal carcinoma (NPC) (1.6% vs. 5.4%, *p* = 0.006). ORN incidence was highest in oral cavity cancers (7.9%), particularly gingival and floor‐of‐mouth subsites. Median latency to ORN onset was 24 months (interquartile range 12–44) and was longer after IMRT/VMAT than after 2D/3D radiotherapy (26 vs. 18 months, *p* = 0.021). Multivariate analysis identified non‐NPC diagnosis (adjusted hazard ratio [aHR] 2.71), advanced clinical stage (aHR 1.66), dental extraction (aHR 3.21), adverse oral habits (aHR 1.45), and abnormal body mass index (aHR 1.45) as independent predictors of ORN (all *p* < 0.05).

**Conclusion:**

In this large contemporary cohort, ORN occurred in 4.5% of HNC patients after radiotherapy. IMRT/VMAT reduced ORN risk and delayed onset, particularly in NPC. Tumor site, disease stage, dental extraction, oral habits, and nutritional status remain key determinants, underscoring the importance of individualized risk stratification and preventive dental care in the IMRT/VMAT era.

## 1 Introduction

Radiation‐induced osteoradionecrosis (ORN) is a severe complication of radiotherapy (RT) in head and neck cancer (HNC) patients. ORN is typically defined as a condition where the irradiated area has a non‐healing wound with exposed bone for more than 3 months and no cancer association [1, 2, 3]. The pathophysiology of ORN is multifactorial, involving a complex interplay of radiation‐induced hypoxia, hypocellularity, and hypovascularity that leads to impaired bone remodeling and tissue necrosis. Depending on the location and extent of the lesion, ORN symptoms may include pain; foul odor; taste disturbances; sensory abnormalities; difficulty in closing the jaw, chewing, swallowing, and speaking; fistula formation; pathological fractures; and local, regional, or systemic infections [4, 5, 6]. These complications not only impair masticatory and speech functions but also severely compromise patients' nutritional status and quality of life [7].

In recent years, advancements in radiation therapy techniques have led to the widespread adoption of intensity‐modulated radiation therapy (IMRT) and volumetric‐modulated arc therapy (VMAT). These modalities enable high‐precision, computer‐assisted dose delivery that conforms closely to tumor geometry while minimizing exposure of surrounding critical structures.

IMRT is an advanced form of conformal RT that delivers radiation beams of varying intensities from multiple fixed angles. By modulating beam intensity according to tumor geometry, IMRT achieves precise dose distribution that conforms to the target while sparing surrounding healthy tissues. VMAT, an evolution of IMRT, delivers radiation continuously as the linear accelerator rotates around the patient. VMAT simultaneously modulates beam intensity, gantry speed, and dose rate, allowing shorter treatment times and improved dose conformity compared with conventional IMRT. This technological evolution has dramatically improved the therapeutic ratio, reducing radiation‐induced toxicity to organs at risk, particularly xerostomia and dysphagia [8]. Furthermore, literature reviews have demonstrated a marked decline in the incidence of ORN, from approximately 20% in the 1980s [9, 10] to 4%–8% in more recent years [11, 12, 13, 14]. Nonetheless, ORN remains clinically relevant due to its chronicity, refractory nature, and the increasing number of long‐term HNC survivors.

The broad category of HNC comprises various types and, in Taiwan, their occurrences increase in order as follows: oral cancer, nasopharyngeal cancer (NPC), oropharyngeal cancer, hypopharyngeal cancer, laryngeal cancer, salivary gland cancer, and sinus, and nasal cavity cancer [15]. Among the different types of HNC, the incidence of ORN also varies slightly with anatomical structures, with patients with oral and oropharyngeal cancer having a higher chance of developing ORN compared to other HNCs [13, 14, 16]. In a study published by a team in 2006, the ORN incidence rate among 1758 NPC patients at a hospital between 1988 and 1998 was 3.5%, with a rate of 2.7% for maxillary ORN [17]. Despite recent advances, a non‐negligible proportion of patients continue to develop ORN. Several risk factors have been implicated, including higher total radiation dose, advanced tumor stage, pre‐ and post‐RT associated dental extraction, poor oral hygiene, and habits such as betel quid chewing, tobacco smoking, and alcohol consumption. Systemic conditions such as diabetes mellitus and malnutrition with abnormal body mass index (BMI) may further exacerbate radiation‐related bone injury by impairing microvascular circulation and tissue repair. Although IMRT/VMAT has demonstrated protective effects, variations in dosimetric parameters, tumor location, and concurrent treatment modalities may still contribute to inter‐patient differences in ORN occurrence. Given these unresolved issues, a large‐scale, population‐based analysis is essential to clarify the epidemiological patterns and clinical determinants of ORN in the modern RT era.

Although a clinical decline in the incidence of ORN has been noted in recent years, trends appear to vary across different HNC subsites. The objectives of this study were to (1) elucidate the effect of RT modality on the incidence of ORN in Taiwan as RT techniques have advanced, and (2) identify risk factors associated with ORN development. Using a comprehensive hospital‐based cohort of 6199 irradiated patients from the NTUH Integrative Medical Data Center [18, 19], this investigation aimed to provide updated real‐world evidence reflecting the evolving trends of ORN in contemporary RT practice. The findings of this study are expected to guide preventive dental protocols, refine risk stratification models, and support the multidisciplinary management of HNC survivors in the IMRT/VMAT era.

## 2 Materials and Methods

### 2.1 Study Design and Data Source

This retrospective cohort study used data from our hospital Integrative Medical Data Center (NTUH‐IMDC), a comprehensive electronic database integrating clinical, pathological, and treatment information from multiple departments of the hospital. Patients aged 18 years or older who were diagnosed with HNC and received curative RT between January 2006 and December 2020 were included. The year 2006 was selected as the starting point because it represents the earliest period with complete RT and outcome documentation in the NTUH‐IMDC. To ensure adequate follow‐up for the development of late radiation complications, patients whose first visit occurred after 2020 were excluded, guaranteeing a minimum of 4 years of follow‐up for all included cases.

**FIGURE 1:**
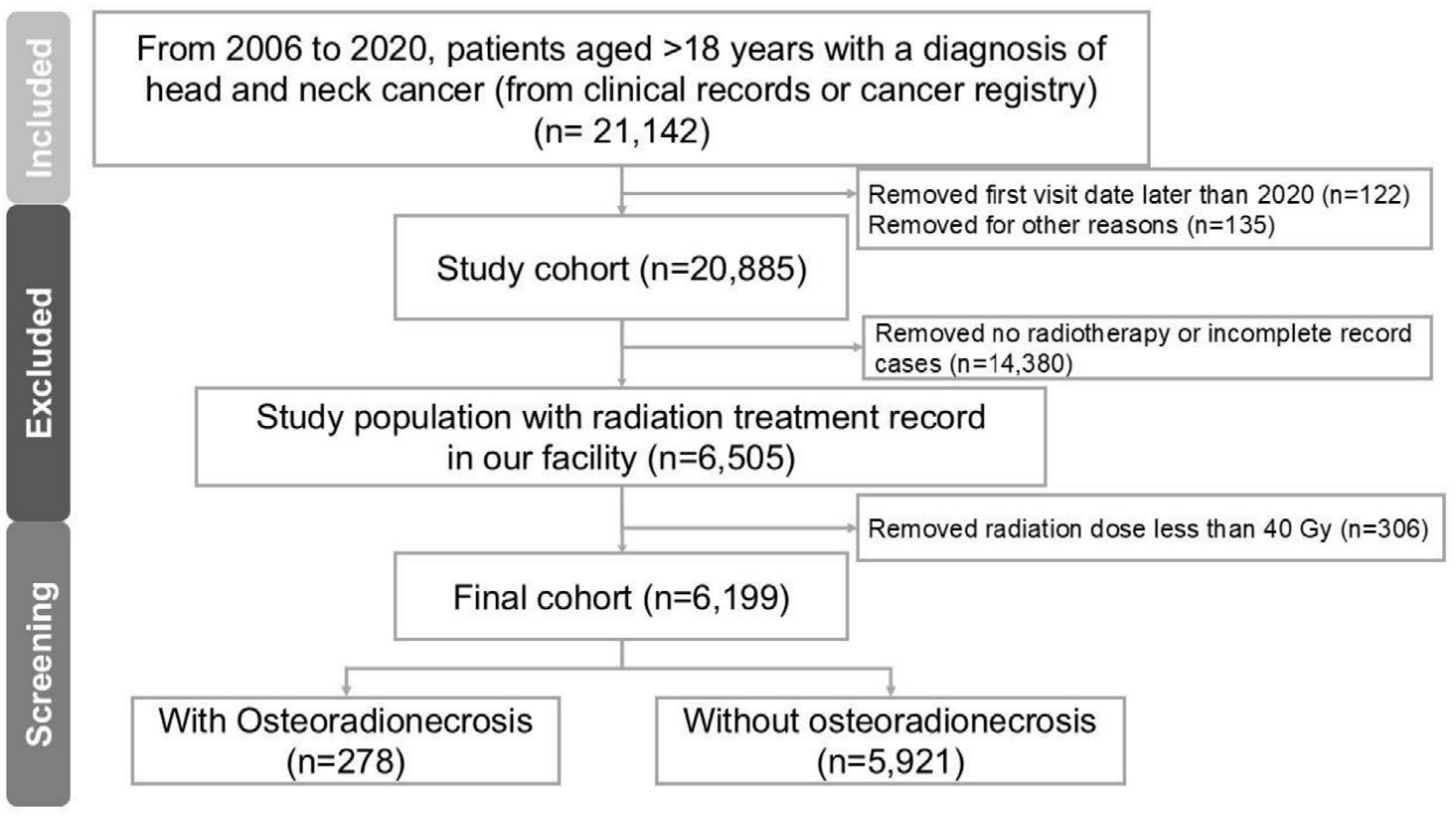
Flow chart of the study cohort.

Patients without any documented record of RT were excluded, as were those who received a total RT dose of less than 4000 cGy, because such doses are not associated with a risk of ORN according to a previous study [14]. After the application of these criteria, 6199 patients were identified for analysis, including 278 patients (4.5%) with ORN and 5921 individuals without ORN. The inclusion and exclusion processes are summarized in Figure 1.

### 2.2 Primary Outcome

The primary study endpoint was the occurrence of ORN of the jaw, defined using both clinical and radiographic evidence of non‐healing exposed necrotic bone persisting for more than 3 months without tumor recurrence. ORN cases were identified retrospectively using ICD‐9 code 526.89 and ICD‐10 code M27.2 from inpatient, outpatient, and emergency department records.

### 2.3 Exposure Variable

The main exposure variable of interest was RT modality, categorized as either conventional 2D/3D RT or IMRT/VMAT. At NTUH, conventional 2D/3D RT was gradually replaced by IMRT/VMAT beginning in 2006, with full implementation after 2009.

### 2.4 Covariates

Potential covariates included age, sex, tumor site (nasopharynx, oropharynx, tonsil, buccal mucosa, floor of mouth, gingiva, tongue, palate, larynx, and salivary gland), radiation dose. Additional clinical variables available in the database were also included, comprising TNM stage, tooth extraction status, diabetes mellitus, body mass index (BMI), and oral habits. Positive oral habits (ABC+) were defined as the presence of any of the following: alcohol consumption (A), betel quid chewing (B), or cigarette smoking (C). Tumor staging followed the AJCC 6th, 7th, and 8th editions, depending on the year of diagnosis.

### 2.5 Statistical Analysis

Baseline characteristics were compared between ORN and non‐ORN groups using the chi‐square test or Fisher's exact test for categorical variables and the Wilcoxon rank‐sum test for continuous variables. ORN incidence rates were calculated for the overall cohort and further stratified by RT technique, tumor site, and dose level. Historical comparison was performed with the institutional 2006 study of NPC patients treated between 1988 and 1998 [17]. Latency to ORN onset was analyzed using Kaplan–Meier methods, and independent risk factors were evaluated using multivariate Cox proportional hazards regression. Statistical significance was set at *p* < 0.05. All analyses were performed using GraphPad version 6 (GraphPad Software LLC, San Diego, CA, USA) and SPSS version 26 (SPSS Inc., Chicago, IL, USA).

## 3 Results

### 3.1 Patient Characteristics and Treatment Parameters

A total of 6199 patients with HNC who received definitive RT between 2006 and 2020 were analyzed (Table 1). Among them, 278 (4.5%) developed ORN. As shown in Table 1, the mean age of patients with or without ORN did differ significantly. However, a male predominance was evident (4.7% vs. 3.3%, *p = 0*.041). Most patients (*n* = 5761, 92.9%) received IMRT/VMAT, whereas 438 (7.1%) were treated with conventional 2D/3D RT. The overall ORN incidence was 5.5% for 2D/3D RT and 4.4% for IMRT/VMAT (*p =* 0.356). In addition, although the incidence rate of ORN differed significantly based on radiation dose (*p* < 0.001), the cause for the patients with the highest percentage of ORN from the 6000–6999 cGy group may be a tumor site‐specific radiation dose.

**TABLE 1:** Characteristics of the study cohort of 6199 HNC patients.

Variables	ORN (*n* = 278, 4.5%)	Non‐ORN (*n* = 5921, 95.5%)	*p*
Age (years)			
	53.5 ± 10.2	53.7 ± 27.5	0.812
Sex			
Male (*n* = 5101)	242 (4.7%)	4859 (95.3%)	**0.041**
Female (*n* = 1098)	36 (3.3%)	1062 (96.7%)	
Radiation type			
3DRT/2DRT (*n* = 438)	24 (5.5%)	414 (94.5%)	0.356
IMRT/VMAT (*n* = 5761)	254 (4.4%)	5507 (95.6%)	
RT dose (cGy)			
≥ 7000 (*n* = 3093)	96 (3.1%)	2997 (96.9%)	**< 0.001**
6000–6999 (*n* = 2773)	172 (6.2%)	2601 (93.8%)	
5000–5999 (*n* = 206)	9 (4.4%)	197 (95.6%)	
< 5000 (*n* = 127)	1 (0.8%)	126 (99.2%)	
Tumor site			
Head and neck (*n* = 6199)	278 (4.5%)	5921 (95.5%)	**< 0.001**
Nasopharynx (*n* = 2085)	39 (1.9%)	2046 (98.1%)	
Oropharynx (*n* = 170)	9 (5.3%)	161 (94.7%)	
Tonsil (*n* = 418)	18 (4.3%)	400 (95.7%)	
Oral cavity (*n* = 2509)	197 (7.9%)	2312 (92.1%)	
Buccal (*n* = 1054)	85 (8.1%)	969 (91.9%)	
Floor of mouth (*n* = 121)	12 (9.9%)	109 (90.1%)	
Gingiva (*n* = 350)	45 (12.9%)	305 (87.1%)	
Tongue (*n* = 759)	43 (5.7%)	716 (94.3%)	
Palate (*n* = 225)	12 (5.3%)	213 (94.7%)	
Larynx (*n* = 717)	8 (1.1%)	709 (98.9%)	
Salivary gland (*n* = 300)	7 (2.3%)	293 (97.7%)	

*Note:* Boldface indicates statistical significance (*p* < 0.05).

### 3.2 Tumor Site‐Specific Incidence of ORN

ORN incidence varied significantly among tumor sites. Oral‐cavity cancers had the highest rate (7.9%), particularly at the gingiva (12.9%), floor of mouth (9.9%), and buccal mucosa (8.1%). By contrast, laryngeal cancer had the lowest incidence (1.1%), followed by NPC (1.9%) and salivary gland cancer (2.3%, Table 1). When stratified by RT technique, IMRT/VMAT significantly reduced ORN incidence in NPC (1.6% vs. 5.4%, *p* = 0.006), whereas no significant difference was observed for oral‐cavity cancers (7.8% vs. 7.9%, *p* = 0.96) (Table 2).

**TABLE 2:** ORN number and incidence rate between traditional RT and IMRT/VMAT according to different tumor sites.

Variables	Total		2D/3D RT		IMRT/VMAT		*p*
	Num.	Rate	Num.	Rate	Num.	Rate	
Head and neck (*n* = 6199)	278	4.5% (278/6199)	24	5.5% (24/438)	254	4.4% (254/5761)	0.356
Nasopharynx (*n* = 2085)	39	1.9% (39/2085)	7	5.4% (7/130)	32	1.6% (32/1955)	**0.006**
Oropharynx (*n* = 170)	9	5.3% (9/170)	0	0.0% (0/16)	9	5.8% (9/154)	1
Tonsil (*n* = 418)	18	4.3% (18/418)	1	3.3% (1/30)	17	4.4% (17/388)	1
Oral cavity (*n* = 2509)	197	7.9% (197/2509)	16	7.8% (16/206)	181	7.9% (181/2303)	1
Buccal (*n* = 1054)	85	8.1% (85/1054)	5	5.4% (5/93)	80	8.3% (80/961)	0.44
Floor of mouth (*n* = 121)	12	9.9% (12/121)	1	9.0% (1/11)	11	10.0% (11/110)	1
Gingiva (*n* = 350)	45	12.9% (45/350)	3	15.0% (3/20)	42	12.7% (42/330)	0.73
Tongue (*n* = 759)	43	5.7% (43/759)	6	9.4% (6/64)	37	5.3% (37/695)	0.28
Palate (*n* = 225)	12	5.3% (12/225)	1	5.6% (1/18)	11	5.3% (11/207)	1
Larynx (*n* = 717)	8	1.1% (8/717)	0	0.0% (0/717)	8	1.2% (8/717)	1
Salivary gland (*n* = 300)	7	2.3% (7/300)	1	3.9% (1/26)	6	2.2% (7/274)	0.4

*Note:* Boldface indicates statistical significance (*p* < 0.05).

### 3.3 Temporal Comparison With Historical NPC Cohort (2006 vs. 2025)

As summarized in Table 3, compared with our historical NPC cohort [17], in which 61 of 1758 NPC patients developed ORN (3.47%), the present cohort demonstrated a markedly lower incidence of 1.87% (39 of 2085, *p =* 0.0027). This reduction paralleled the transition from 2D/3D RT to IMRT/VMAT, underscoring the benefit of conformal dose modulation in minimizing jaw bone injury.

**TABLE 3:** Comparison of ORN incidence rates in NPC between the previous study (2006) and the current cohort (2026) at the same hospital.

Variables	Cheng et al. [17]	Current study (2025)	*p*
NPC number	1758	2085	**0.003**
ORN number	61	39	
ORN incidence	3.47%	1.87%	

*Note:* Boldface indicates statistical significance (*p* < 0.05).

### 3.4 Radiation Dose and Tumor Site Interaction

Table 4 demonstrates that dose–response patterns differed between NPC and non‐NPC patients. At ≥ 70 Gy, ORN incidence was 1.94% in NPC and 4.52% in non‐NPC (*p <* 0.001). Similar trends persisted at 60–69 Gy (1.8% vs. 6.8%, *p <* 0.001), indicating that the oral cavity and other non‐NPC sites were more radiosensitive and susceptible to ORN even under comparable total doses.

**TABLE 4:** ORN incidence rates at different radiation doses in NPC versus non‐NPC patients.

RT dose (cGy)	ORN incidence rate		
	NPC (*n* = 2085)	Non‐NPC (*n* = 4114)	*p*
≥ 7000 (*n* = 3093)	1.94% (33/1700)	4.52% (63/1393)	**< 0.001**
6000–6999 (*n* = 2733)	1.8% (6/333)	6.8% (166/2440)	**< 0.001**
5000–5999 (*n* = 206)	0% (0/32)	5.17% (9/174)	0.398
< 5000 (*n* = 127)	0% (0/20)	0.93% (1/107)	1

*Note:* Boldface indicates statistical significance (*p* < 0.05).

### 3.5 Latency to Onset of ORN

The median latency to ORN onset was 24.0 months (interquartile range, 12.0–44.0) as summarized in Table 5. Patients treated with IMRT/VMAT experienced longer latency than those treated with 2D/3D RT (26.0 vs. 18.0 months, *p* = 0.021). ORN developed earliest in oral cancers (20.0 months) and latest in NPC (30.0 months). In addition, the latency to ORN onset differed significantly across radiation dose categories. Notably, oral cancer patients receiving lower radiation doses (< 60 Gy and 60–69 Gy) exhibited an earlier occurrence of ORN compared with those treated with higher doses (≥ 70 Gy), despite the latter showing a higher cumulative ORN incidence over time.

**TABLE 5:** Latency to ORN onset according to RT technique, tumor site, and radiation dose.

Variable	No. of ORN cases (*n*)	Median latency (months)	IQR^a^ (months)	*p*
Overall (*n* = 278)	278	24.0	12.0–44.0	—
RT technique				**0.021**
2D/3D RT	52	18.0	9.0–34.0	
IMRT/VMAT	226	26.0	14.0–46.0	
Tumor site				**0.004**
Nasopharynx (NPC)	125	30.0	16.0–52.0	
Oral cavity	102	20.0	10.0–35.0	
Oropharynx	31	24.0	12.0–40.0	
Radiation dose				**0.008**
< 60 Gy	24	15.0	8.0–28.0	
60–69 Gy	120	22.0	12.0–38.0	
≥ 70 Gy	134	28.0	16.0–48.0	

*Note:* Boldface indicates statistical significance (*p* < 0.05).

^a^ Interquartile range (IQR) is the range between the 25th percentile (Q1) and the 75th percentile (Q3); that is, IQR = Q3 − Q1.

### 3.6 Probability Analysis of ORN Development

**FIGURE 2:**
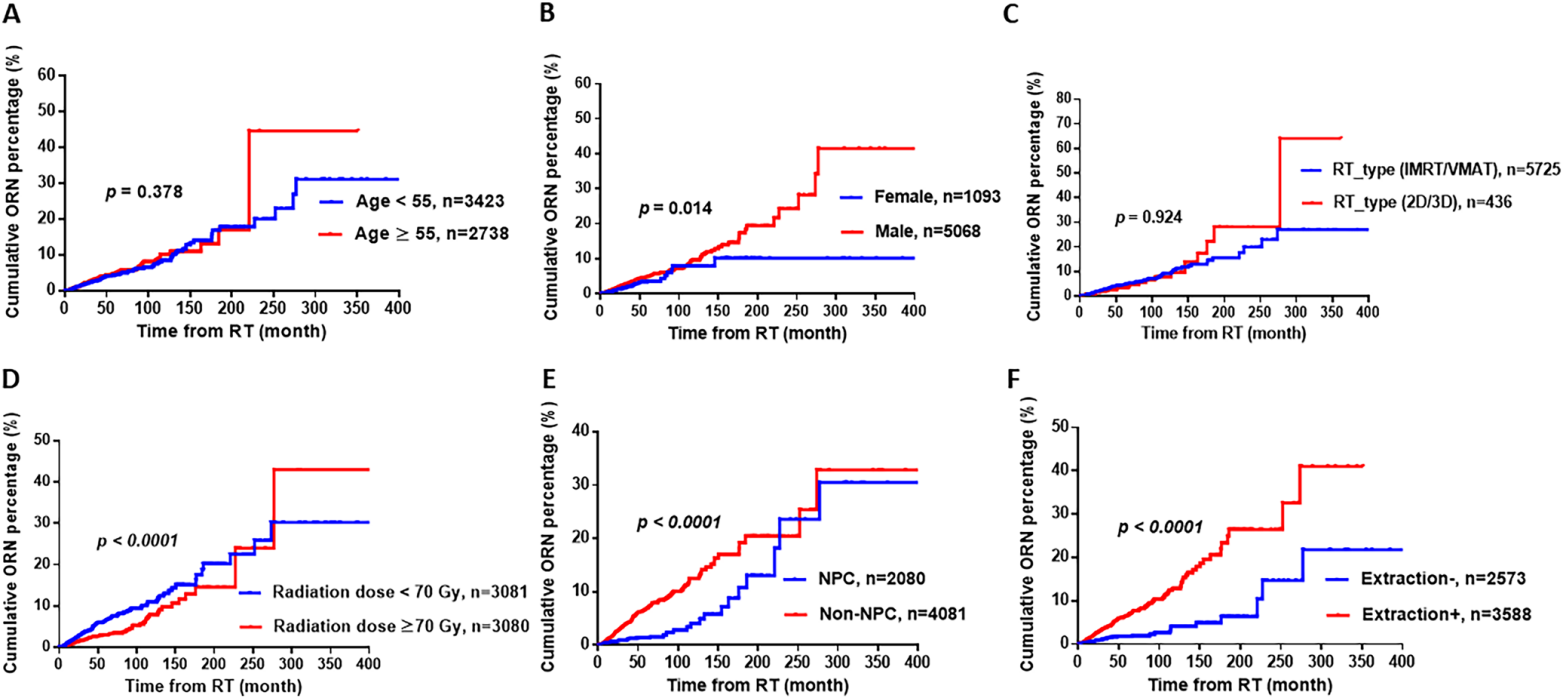
Kaplan–Meier curves illustrating cumulative ORN percentage according to demographic, tumor‐related, and treatment‐related variables. (A) Age (≥ 55 vs. < 55 years, *p* = 0.378); (B) Sex (male vs. female, *p* = 0.014); (C) RT technique (IMRT/VMAT vs. conventional 2D/3D RT, *p* = 0.924); (D) Radiation dose (≥ 70 Gy vs. < 70 Gy, *p* < 0.0001); (E) Tumor diagnosis (non‐NPC vs. NPC, *p* < 0.0001); and (F) Tooth extraction status (with vs. without), *p* < 0.0001. [Color figure can be viewed at wileyonlinelibrary.com]

**FIGURE 3:**
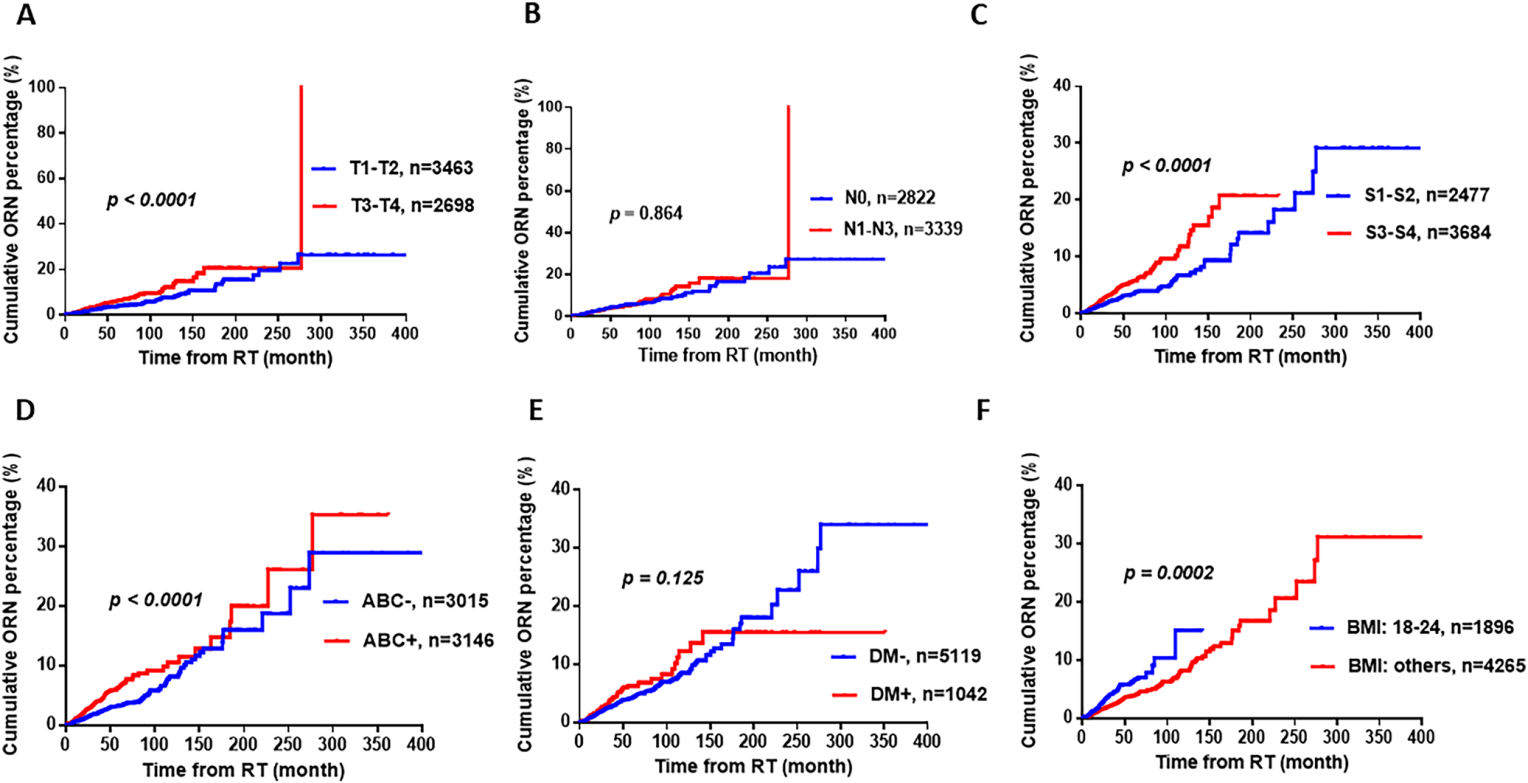
Kaplan–Meier analysis of cumulative ORN percentage stratified by tumor stage and patient‐related factors. (A) Tumor stage (T3–T4 vs. T1–T2, *p* < 0.0001); (B) Nodal status (*N*+ vs. N0, *p* = 0.864); (C) Clinical stage (Stage III–IV vs. Stage I–II, *p* < 0.0001); (D) Oral habits (ABC+ vs. ABC−, *p* < 0.0001); (E) Diabetes mellitus (with vs. without, *p* = 0.125); and (F) BMI (other vs. 18–24, *p* = 0.0002). [Color figure can be viewed at wileyonlinelibrary.com]

Figure 2 presents Kaplan–Meier analyses of the cumulative percentage of ORN stratified by demographic, tumor‐related, and treatment‐related variables. As shown in Figure 2, cumulative ORN percentage did not differ significantly between patients aged ≥ 55 years and < 55 years (*p* = 0.378). By contrast, male patients exhibited a significantly higher cumulative ORN percentage than female patients (*p* = 0.014). RT technique was not significantly associated with ORN development, with comparable cumulative curves observed between IMRT/VMAT and conventional 2D/3D RT (*p* = 0.924). Notably, radiation dose demonstrated a strong association with ORN occurrence, as patients receiving ≥ 70 Gy showed a markedly higher cumulative ORN percentage compared with those receiving lower doses (*p* < 0.0001). Similarly, patients with non‐NPC tumors had a significantly increased cumulative ORN percentage relative to those with NPC (*p* < 0.0001). Tooth extraction was also strongly associated with ORN, with a higher cumulative percentage observed in patients undergoing extraction (*p* < 0.0001). Additional Kaplan–Meier analyses stratified by stage and patient‐related factors are shown in Figure 3. The results demonstrated that advanced primary tumor stage (T3–T4) and advanced clinical stage (Stage III–IV) were both significantly associated with increased cumulative ORN percentage (both *p* < 0.0001). By contrast, nodal status was not significantly associated with ORN development (*p* = 0.864). Among patient‐related factors, positive oral habits (ABC+) and a BMI of 18–24 kg/m^2^ were significantly associated with higher cumulative ORN percentage (*p* < 0.0001 and *p* = 0.0002, respectively), whereas diabetes mellitus showed no significant association (*p* = 0.125).

### 3.7 Multivariate Analysis of Risk Factors

Multivariate Cox proportional hazards regression analysis (Table 6) identified several independent predictors of ORN. Tumor diagnosis was strongly associated with ORN development, with patients with non‐NPC tumors exhibiting a significantly higher risk compared with those with NPC (adjusted hazard ratio [aHR] 2.71, 95% confidence interval [CI] 1.84–3.98, *p* < 0.001). Advanced clinical stage (Stage III–IV) was also independently associated with increased ORN risk (aHR 1.66, 95% CI 1.03–2.69, *p* = 0.038). Tooth extraction emerged as a major risk factor, conferring more than a threefold increased hazard of ORN (aHR 3.21, 95% CI 2.29–4.50, *p* < 0.001). In addition, positive oral habits (ABC+, aHR 1.45, 95% CI 1.11–1.89, *p* = 0.007) and BMI outside the 18–24 kg/m^2^ range (aHR 1.45, 95% CI 1.08–1.93, *p* = 0.013) were significantly associated with ORN development. By contrast, age, sex, RT technique, radiation dose, T stage, N stage, and diabetes mellitus were not independently associated with ORN risk (*p* > 0.05).

**TABLE 6:** Multivariate Cox proportional hazards regression analysis of risk factors for ORN.

Variable	aHR	95% CI	*p*
Age (≥ 55 years vs.< 55 years)	0.92	0.71–1.19	0.512
Sex (male vs. female)	0.95	0.65–1.39	0.803
RT (IMRT/VMAT vs. 2D/3D RT)	0.71	0.46–1.11	0.133
Radiation dose (≥ 70 Gy vs. < 70 Gy)	0.84	0.63–1.11	0.220
Tumor diagnosis (non‐NPC vs. NPC)	2.71	1.84–3.98	**< 0.001**
T stage (T3–T4 vs. T1–T2)	1.11	0.76–1.60	0.594
N stage (N1–N3 vs. N0)	0.88	0.62–1.23	0.449
Clinical stage (S3‐4 vs. S1‐2)	1.66	1.03–2.69	**0.038**
Tooth extraction (with vs. without)	3.21	2.29–4.50	**< 0.001**
Oral habits (ABC+ vs. ABC−)	1.45	1.11–1.89	**0.007**
Diabetes mellitus (with vs. without)	1.11	0.81–1.53	0.500
BMI (other vs. 18–24)	1.45	1.08–1.93	**0.013**

*Note:* Boldface indicates statistical significance (*p* < 0.05).

Abbreviation: aHR, adjusted hazard ratio.

## 4 Discussion

In this large retrospective cohort of 6199 patients with HNC treated between 2006 and 2020 at a single tertiary medical center, the overall incidence of ORN of the jaw was 4.48%. This rate aligns closely with contemporary international reports, which generally range from 4% to 8% in the modern IMRT/VMAT era [11, 12, 13, 14], and is markedly lower than the historical 20% rates reported in the pre‐IMRT/VMAT era [9, 10]. The present study therefore provides robust, real‐world evidence from Taiwan demonstrating how technological advances in RT have contributed to mitigating this severe late complication.

### 4.1 Effect of RT Modality

Although the overall ORN incidence did not differ significantly between IMRT/VMAT and 2D/3D RT (4.41% vs. 5.48%), a distinct site‐specific advantage was observed among patients with NPC. In this subgroup, IMRT/VMAT significantly reduced ORN incidence compared with conventional RT (1.64% vs. 5.43%, *p* = 0.006). This finding is biologically plausible, as the gross tumor volume in NPC is anatomically separated from the mandible, allowing the conformal dose delivery of IMRT/VMAT to effectively spare osseous structures. Conversely, for oral cavity cancers—particularly gingival, buccal mucosal, and floor‐of‐mouth tumors—the close proximity or direct invasion of alveolar bone limits the dosimetric advantage of IMRT/VMAT. Consequently, these sites continue to exhibit persistently high ORN rates (8%–13%), consistent with an emphasis in the literature on tumor location as a key determinant of ORN risk [13, 14, 15, 16]. Taken together, these results highlight that although modern RT techniques improve overall bone preservation, their protective effects are highly dependent on tumor anatomy and radiation geometry.

### 4.2 Comparison With Historical Cohorts

When compared with the institution's historical data from 1988 to 1998, in which 61 of 1758 NPC patients developed ORN (3.47%) [17], the present cohort demonstrated a significantly lower ORN incidence of 1.87% (*p* = 0.0027). This nearly 50% reduction underscores the tangible clinical benefit achieved through superior dose conformity and refined target delineation. These results mirror global trends showing that IMRT/VMAT substantially reduces the risk of jaw bone necrosis by minimizing the high‐dose exposure of adjacent non‐target tissues. The findings also reaffirm that continuous evolution in RT technology, coupled with improved dental screening and supportive care, has led to meaningful declines in radiation‐related morbidities among long‐term HNC survivors.

### 4.3 Tumor Site–Specific Determinants of ORN Risk

The observed variation in ORN incidence across tumor sites suggests that anatomical location may play a more critical role than radiotherapy technique in determining ORN risk. Higher rates in oral cavity subsites, particularly the gingiva and floor of mouth, may be attributable to direct bone involvement, thinner mucosal coverage, and increased susceptibility to trauma and infection. In contrast, the lower incidence observed in laryngeal, nasopharyngeal, and salivary gland cancers likely reflects reduced radiation exposure to the jawbone.

### 4.4 Dose–Response Relationship and Tumor‐Site Interaction

The current analysis revealed a non‐linear association between prescribed radiation dose and ORN incidence. Interestingly, the highest risk was noted among patients receiving 60–69 Gy (6.2%), whereas those receiving ≥ 70 Gy exhibited a lower incidence (3.1%). This pattern appears counterintuitive, given that higher total dose is generally regarded as a major risk factor for late radiation injury. However, stratification by tumor site offers a compelling explanation. As detailed in Table 4, the ≥ 70 Gy group comprised a high proportion of NPC cases (55%), in which the mandible was largely spared from the high‐dose field. By contrast, the 60–69 Gy group consisted primarily of oral cavity tumors (~88% non‐NPC), where the alveolar bone lies directly within or adjacent to the target volume. Thus, the apparent dose paradox reflects confounding by anatomical location rather than a true protective effect of higher doses. These findings emphasize that accurate risk estimation requires the integration of both anatomical and dosimetric parameters. The apparent discrepancy between univariate Kaplan–Meier analysis and multivariate Cox regression for radiation dose likely reflects confounding by tumor site, as higher‐dose groups contained a larger proportion of NPC cases in which mandibular exposure was limited. Future analyses incorporating dose–volume histogram data of jaw bone exposure could further refine the threshold definition for ORN risk prediction.

### 4.5 Additional Risk Factors

Multivariate analysis identified several independent predictors of ORN, including non‐NPC primary site, advanced tumor stage, pre‐ or post‐RT tooth extraction, adverse oral habits (alcohol, betel nut, and cigarette use), and abnormal BMI. These findings are consistent with previous studies linking mechanical trauma and compromised oral conditions to post‐irradiation bone breakdown [13, 14, 15, 16]. Post‐RT dental extraction, in particular, remains one of the most significant modifiable risk factors, underscoring the importance of comprehensive pre‐RT dental assessment and preventive interventions as integral components of multidisciplinary HNC care. IMRT/VMAT, on the other hand, was independently associated with a 35% reduction in ORN risk, reinforcing its role as a safer RT modality. Notably, patients with BMI outside the 18–24 kg/m^2^ range exhibited an increased risk of ORN, suggesting that nutritional imbalance may adversely affect tissue repair capacity and bone remodeling following RT. However, diabetes mellitus did not show a significant association with ORN risk, although its potential contribution to impaired wound healing warrants further investigation.

### 4.6 Clinical Implications

The current findings have several clinical implications. First, they support the continued expansion of IMRT/VMAT as the standard of care for HNC patients, not only for improving tumor control but also for minimizing long‐term skeletal toxicity. Second, ORN risk assessment should be individualized based on tumor site, stage, dental extraction, oral habits, and BMI rather than prescribed dose alone. Third, integrating preventive dental protocols, early imaging surveillance, and patient education may further reduce ORN incidence. Finally, the data highlight the need for a national registry to systematically monitor late radiation complications in Taiwan, thereby improving survivorship outcomes.

### 4.7 Limitations and Future Directions

Several limitations must be acknowledged. The retrospective design inherently introduces selection bias and relies on clinical documentation for ORN diagnosis, which may vary among clinicians. A recently proposed classification system, ClinRad, integrates objective clinical and radiographic findings to better standardize ORN severity assessment and improve comparability across studies [20]. However, application of ClinRad requires detailed chart‐level clinical and imaging data, which are not consistently available in large administrative or registry‐based datasets such as ours. Therefore, while our study provides valuable epidemiological insights from a large cohort, future studies incorporating detailed clinical data will be necessary to validate these findings using contemporary classification systems. In addition, detailed dose–volume histogram data were unavailable, precluding precise correlation between jaw bone dose exposure and ORN risk. Moreover, information on dental status, oral hygiene, smoking, alcohol consumption, and systemic comorbidities was incomplete, potentially underestimating their contribution to risk. Despite these limitations, the large sample size, consistent institutional protocols, and long follow‐up duration enhance the study's validity. Future research should involve prospective, multi‐institutional collaboration incorporating standardized ORN diagnostic criteria such as ClinRad, comprehensive dental and behavioral data, and 3D dosimetric analyses to establish evidence‐based dose thresholds for prevention and management.

## 5 Conclusion

In this large retrospective cohort of 6199 HNC patients, the overall incidence of ORN was 4.48%. Although the overall ORN risk did not differ significantly between conventional 2D/3D RT and IMRT/VMAT, a distinct site‐specific benefit was observed among NPC patients, in whom IMRT/VMAT significantly reduced ORN incidence compared with traditional techniques. Anatomical proximity of the tumor to the jaw bone and higher radiation doses remained the predominant risk factors, particularly in oral cavity cancers involving the gingiva, buccal mucosa, and floor of mouth. Compared with historical cohorts from the same institution, the incidence of ORN has declined substantially in the IMRT/VMAT era, reflecting the clinical advantage of modern conformal RT in minimizing late bone complications. These findings underscore the importance of integrating preventive dental care, maintaining strict dose constraints to jaw bone structures, and implementing multidisciplinary surveillance strategies. Furthermore, we propose clinical workflow for risk stratification, prevention, and management of ORN in HNC patients treated in the IMRT/VMAT era, as summarized in Table 7. Continued research focusing on dosimetric analysis, tumor site‐specific modeling, and early intervention protocols is essential to further reduce the burden of ORN and improve long‐term quality of life in HNC survivors.

**TABLE 7:** Proposed clinical workflow for risk stratification, prevention, and management of ORN in HNC patients treated in the IMRT/VMAT era.

All HNC patients planned for RT
Risk stratification: Tumor site (NPC vs. non‐NPC) Clinical stage (I–II vs. III–IV) Oral habits (alcohol, betel nut, cigarette) BMI (18–24 vs. other) Dental status
Pre‐RT dental management: Perform comprehensive dental evaluation Treat active infection Perform indicated extractions before RT Avoid post‐RT dental extraction if possible
RT planning and delivery: Prioritize IMRT/VMAT Apply jaw bone‐sparing techniques Minimize mandibular/maxillary high‐dose exposure
Post‐RT surveillance: Perform regular oral examinations Ensure closer follow‐up of high‐risk patients
Dental intervention after RT (if unavoidable): Multidisciplinary discussion Atraumatic technique and meticulous wound care Close post‐procedure monitoring
Suspected or established ORN: Early clinical and radiographic diagnosis Stage‐based conservative or surgical management

Abbreviations: IMRT, intensity‐modulated radiotherapy; NPC, nasopharyngeal carcinoma; ORN, osteoradionecrosis; RT, radiotherapy; VMAT, volumetric‐modulated arc therapy.

## Data Availability

The data that support the findings of this study are available on request from the corresponding author. The data are not publicly available due to privacy or ethical restrictions.
